# Resistance of White Sapphire and Hot-Pressed Alumina to Collision With Liquid Drops

**DOI:** 10.6028/jres.064A.049

**Published:** 1960-12-01

**Authors:** Olive G. Engel

## Abstract

Fused alumina has been reported to be one of the most promising materials for resistance to erosion due to high-speed collision with liquid drops. In this paper, data are presented that show the resistance of 0.318-cm (0.125-in.)-thick plates of white sapphire and hot-pressed alumina to impingement damage by 0.2-cm-diam waterdrops and mercury drops. The type of damage done to these high-strength ceramics as a result of collision with a mercury drop at high velocity was found to be qualitatively the same as that produced on low-strength plastics as a result of collision with a waterdrop at relatively low velocity.

In collision with mercury drops, the velocity at which damage was first observed was 3.514×10^4^ cm/sec (1,153 ft/sec) for white sapphire and 4.276×10^4^ cm/sec (1,403 ft/sec) for hot-pressed alumina; the difference in the velocities found for the two ceramics is not considered to be significant.

The velocity required to damage these ceramic materials on collision with a waterdrop was not reached experimentally. A theoretical extrapolation suggests that plates of these ceramics of the indicated thickness can be expected to survive collision with a 0.2-cm waterdrop without damage up to a velocity of 33.7×10^4^ cm/sec (11,100 ft/sec). For air at 0 °C, this is equivalent to a Mach Number of 10.

## 1. Introduction

The leading surfaces of objects that fly at high speed through rain are eroded as a result of collision with raindrops. The erosion damage increases in severity as the relative impingement velocity is increased. It has become a major problem in the present era of high-speed flight.

A search for materials that will resist this type of damage has been made for the past 10 years. Experimental testing of materials has been carried out at many laboratories of the aircraft industry. An effort to determine the cause of the damage and the mechanism by which it is produced has been in progress at the National Bureau of Standards; this research has been conducted under the sponsorship of the Nonmetallic Materials Laboratory, Materials Central, Wright Air Development Division.

One of the questions asked at the time that the research program on the mechanism of high-speed rain erosion was initiated was whether a soft rubbery material or a hard rigid material should be sought as the solution to the problem. The answer to this question is now at hand. In practical terms, the answer is that there are rubbery coating materials that can withstand collision with waterdrops up to impingement velocities of roughly 3×10^4^ cm/sec (1,000 ft/sec) but that at higher velocities the solution to the problem must be sought among the hard rigid materials of high strength.

Fused alumina is one of the most promising materials of the latter type for resistance to erosion due to high-speed collision with liquid drops. In this paper results of an investigation of the overall resistance and particular mode of failure of white sapphire and hot-pressed alumina, under high-speed liquid-drop impingement, are presented.

## 2. Materials and Test Method

### 2.1. White Sapphire

White sapphire is a pure, single crystal alpha alumina made by dropping finely powdered alumina through a postmixed oxyhydrogen flame onto the molten cap of a seed crystal supported in an insulating furnace. Crystallization occurs at the interface between the body of the crystal and the molten cap. Under proper operating conditions, the material that crystallizes has the same orientation as the seed crystal. The as-grown crystals are annealed at 1,900 °C (3,452 °F) to remove strains.

Five disk-shaped target plates of white sapphire 1.59 cm (0.625 in.) in diameter and 0.318 cm (0.125 in.) thick were obtained from the Linde Air Products Company. They were crystal clear and had the appearance of high-quality glass. The face of the target plate that would strike the liquid drop was given a high polish by the manufacturer.

The physical properties of white sapphire, taken from data supplied by the manufacturer, are listed in table 1.

### 2.2. Hot-Pressed Alumina

Hot-pressed alumina (gray) differs from polycrystalline alumina (cream white) in that it is heated and pressed in one operation rather than in two. The heating and pressing is done in graphite dies and hot-pressed alumina is gray in color because it picks up a carbon impurity from the dies. The amount of carbon that diffuses into the alumina depends on the pressure, temperature, and time involved in the hot-pressing operation. Hot pressing produces an alumina having a density close to that of white sapphire; there is, however, a considerable amount of variability in the product from lot to lot.

Five plates of this alumina of the same size and shape as the white sapphire plates were obtained from the Norton Company. They were opaque and light gray. The impact face of each plate was given a high polish by the manufacturer by means of diamond grinding and lapping.

Properties of hot-pressed alumina also are listed in table 1.

### 2.3. Liquid-Drop Collision Test

The collision experiments were carried out by Convair, Division of General Dynamics Corporation. The ceramic target plates were fastened with adhesive to metal disks of the same size and were fired against mercury drops and waterdrops at velocities ranging from 2.89×10^4^ cm/sec (948 ft/sec) to 11.88×10^4^ cm/sec (3,898 ft/sec). The metal backing disks kept the ceramic target plates that were shattered by the liquid-drop collisions from falling into pieces.

The drop size specified for the collisions was 0.2-cm diam. The mercury drops were weighed on an analytical balance and the diameter of each drop was calculated from its weight. The mercury drops that struck the target plates ranged from 5 percent above to 1 percent below the specified size. It was estimated by the experimenters that the waterdrops were within ±10 percent of the nominal size.

An attempt was made to distribute the velocities at which the target plates were fired. Closely similar velocities were sometimes unavoidably obtained, however, because the velocity produced by a given weight of powder is affected by temperature variation, amount of casing crimp, and the density of powder pack. The velocities at which the target plates were fired are listed in table 2.

## 3. Observations

The target plates of white sapphire and hot-pressed alumina that had collided with mercury drops and waterdrops were examined with a stereo-microscope. The following observations were made.

*No damage was done to either ceramic by collision with a 0.2-cm-diam waterdrop at the highest collision velocities that were used*, namely, 6.620×10^4^ cm/sec (2,172 ft/sec) for white sapphire and 11.88×10^4^ cm/sec (3,898 ft/sec) for hot-pressed alumina. In collision with 0.2-cm-diam mercury drops, the velocity at which damage was first observed was 3.514×10^4^ cm/sec (1,153 ft/sec) for white sapphire and 4.276×10^4^ cm/sec (1403 ft/sec) for hot-pressed alumina; the difference in the velocities found for the two ceramics is not significant because the velocity given for hot-pressed alumina is the lowest velocity at which collision between a mercury drop and this ceramic occurred.

The first evidence of failure of these high-strength ceramics under high-speed mercury-drop impingement was the formation of cracks that bound a more or less circular undamaged area around the central point or eye of the collision. In white sapphire these cracks were polygonal; in hot-pressed alumina they were circular. These cracks ran deep into the ceramic material. The cracks nearest to the central point of the collision were nearly perpendicular to the surface of the target plate but the outer-lying cracks were inclined at an angle to the surface.

The material of the target plate was broken out of the surface along these cracks and on the side of the cracks away from the central point of the collision, that is, in the direction of the radial flow of the liquid of the drop that impinged. The erosion damage was more severe the higher the velocity at which the collision occurred.

At high-impingement velocities radial cracks formed. They ran out from the center of the collision and extended completely across and through the target plate.

Curved cracks that appear to reflect the curved edge of the target plates also formed when the impingement velocity was high. Cracks of this kind observed in white sapphire were below the surface of the target plate; the curved crack observed in hot-pressed alumina extended to the surface.

The observations of damage caused by collision with mercury drops are detailed in the following sections.

### 3.1. White Sapphire

**Figure 1:**
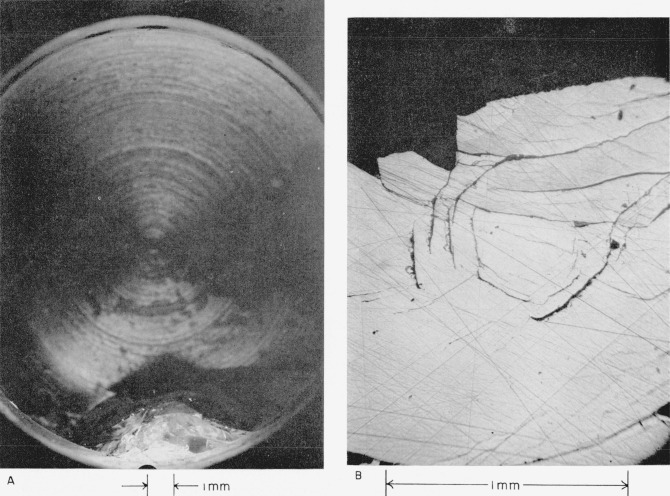
White sapphire target plate No. II that collided with a 0.2-cm mercury drop at a velocity of 3.514×10^4^ cm/sec (1,153 ft/sec). A, View showing extent of damage to the target plate. B, View of the central point of the collision.

The highest velocity at which plate No. II collided is the lowest at which any damage was produced (see table 2). Figure 1A shows that the damage on plate II is restricted to the immediate site of the collision at the edge of the plate. Machining marks in the metal backing plate can be seen through the clear sapphire.

Figure 1B shows that *the eye of the collision consists of an undamaged central area surrounded by an imperfectly developed polygon of short straight cracks that are almost parallel to one another*; possible explanations are given in sections 4.2(a) and 4.2(b). Evidence that these cracks extend down into the target plate in a conchoidal type of fracture is presented below. *Sapphire has been broken out of the surface along the polygonal cracks and on the side of the cracks away from the center of the collision, that is, in the direction of the radial flow of the liquid of the drop that impinged*; a possible cause is discussed in section 4.2(c).

**Figure 2:**
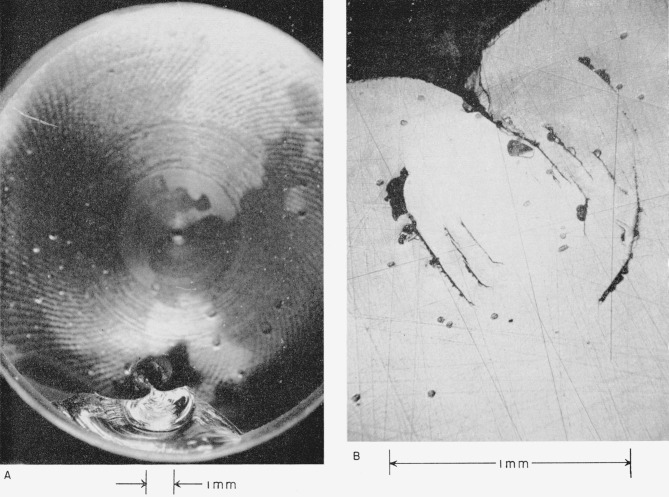
White sapphire target plate No. III that collided with a 0.2-cm mercury drop at a velocity of 3.581×10^4^ cm/sec (1,175 ft/sec). A, View showing extent of damage to the target plate. B, View of the central point of the collision.

Plate No. III collided at a slightly higher velocity (see table 2). Figure 2A shows that the damage is still restricted to the immediate site of the collision close to the edge of the plate; a piece of sapphire was chipped away. Machining marks in the metal backing plate and bubbles in the adhesive can be seen through the sapphire. Figure 2B shows that the eye of the collision is again an imperfectly developed polygon of cracks around an undamaged center. Sapphire is broken out in much the same way as on plate No. II (see above).

**Figure 3:**
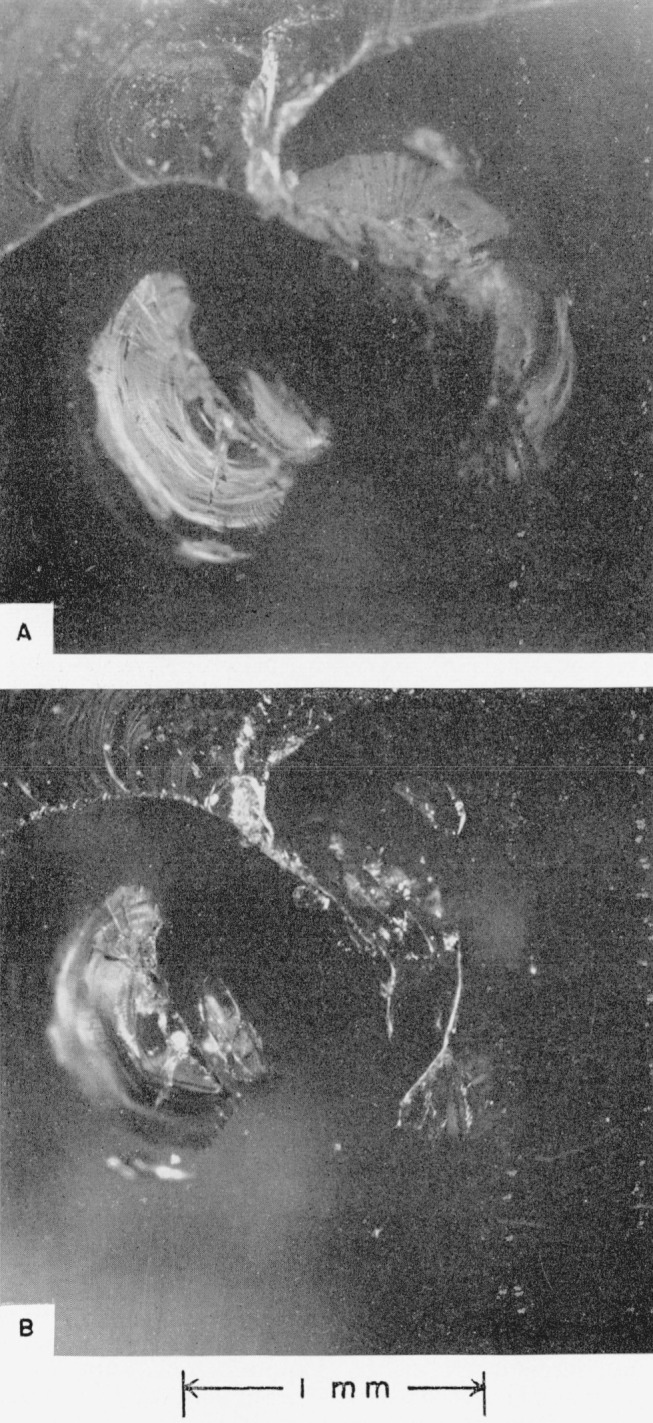
View of the subsurface fractures extending from the surface cracks shown in figure 2B.

*The polygonal cracks extend below the surface in a conchoidal fracture*, as can be seen in the views of the eye of this collision shown in figure 3. These pictures were taken with a different mode of lighting, at lower magnification, and with greater depth of focus. In figure 3A the structure of the subsurface fractures can be seen best but the polygonal cracks on the surface are dim. In figure 3B it can be seen that the subsurface fractures extend from the surface cracks and that, *with increasing depth, they diverge from a line taken through the central point of the collision.*

**Figure 4:**
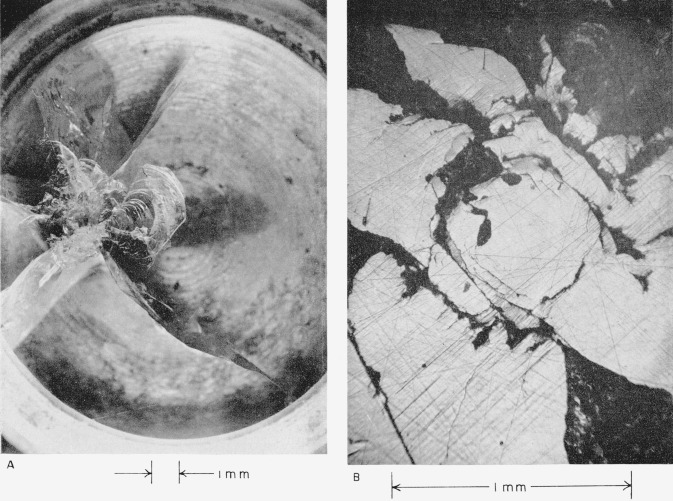
White sapphire target plate No. IV that collided with a 0.2-cm mercury drop at a velocity of 4.420×10^4^ cm/sec (1,450 ft/sec). A, View showing extent of damage to the target plate. B, View of the central point of the collision.

The high velocity at which collision between plate No. IV *and a mercury drop occurred* (see table 2) *resulted in radial cracks that extend completely across and through the thickness of the plate*. They are shown in figure 4A and discussed in section 4.2(d).

Figure 4B shows the eye of this collision. Sapphire has been broken out in much the same way as on plate No. II (see above). The large dark regions are areas where sapphire appears to have been chipped from the surface between intersecting cracks.

**Figure 5:**
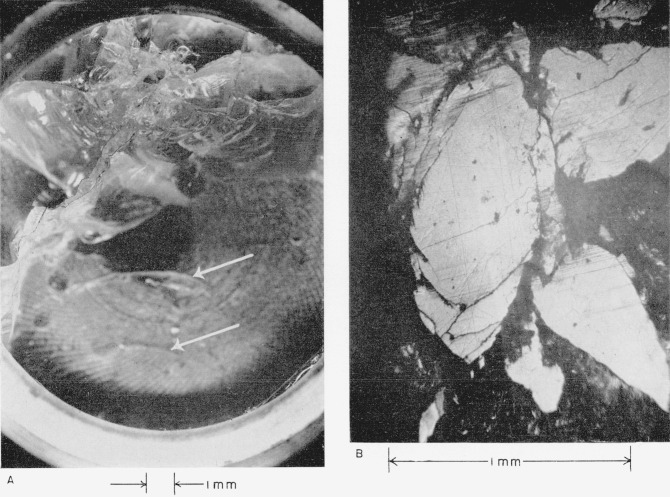
White sapphire target plate No. V that collided with a 0.2-cm mercury drop at a velocity of 4.584×10^4^ cm/sec (1,504 ft/sec). A, View showing extent of damage to the target plate. B, View of the central point of the collision.

Figure 5A shows damage done to plate No. V at the highest velocity at which a white sapphire plate collided with a mercury drop (see table 2). Extensive radial cracks run out from the center of the collision where a considerable amount of sapphire has been chipped out of the surface, apparently between intersecting cracks. *Two semicircular cracks*, *which appear to reflect the curved side of the plate*, *formed as a result of this highest-velocity collision.* They are indicated with arrows in figure 5A and are discussed in section 4.2(e).

Figure 5B shows the eye of this collision.

### 3.2. Hot-Pressed Alumina

**Figure 6:**
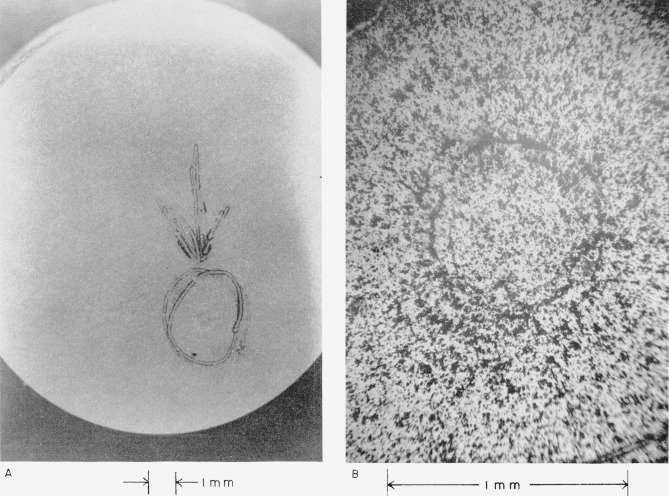
Hot-pressed alumina target plate No. IV that collided with a 0.2-cm mercury drop at a velocity of 4.276×10^4^ cm/sec (1,403 ft/sec). A, View showing extent of damage to the target plate. B, View of the central point of the collision.

Views of damaged hot-pressed alumina target plate No. IV, which collided with a mercury drop (see table 2), are shown in figures 6A and 6B. The experimenters at Convair drew a circle around the damage mark, which they reported was 0.0005 cm (0.0002 in.) deep and 0.071 cm (0.028 in.) in diameter.

*The eye of the collision* (see fig. 6B) *consists of an undamaged central area surrounded by circles of short cracks*. Because the hot-pressed alumina is opaque, damage below the surface cannot be assessed.

**Figure 7:**
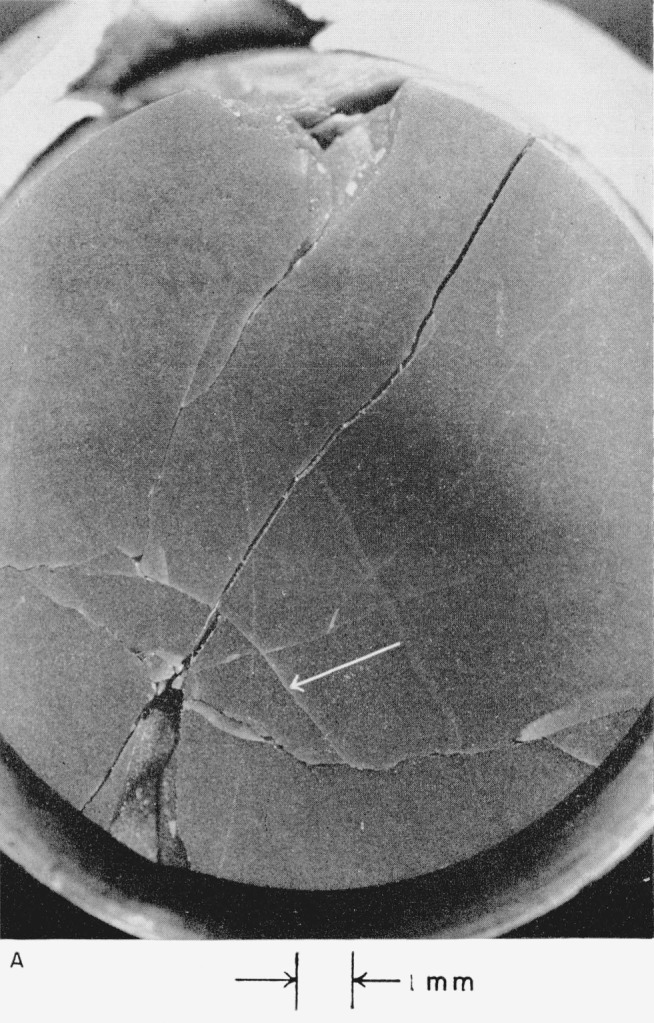

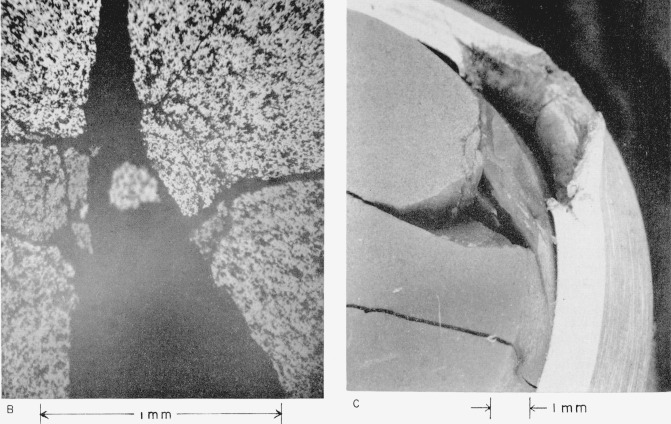
Hot-pressed alumina target plate No. V that collided with 0.2-cm mercury drops at velocities of 4.100×10^4^ cm/sec (1,345 ft/sec) and 4.996×10^4^ cm/sec (1,639 ft/sec). A, View showing extent of damage to the target plate. B, View of the central point of the lower-velocity collision. C, View of the higher-velocity collision site (edge of plate).

Hot-pressed alumina target plant No. V was fired twice. It was chipped by an edge collision at 4.996×10^4^ cm/sec (1,639 ft/sec) and then shattered by collision at 4.100×10^4^ cm/sec (1,345 ft/sec). See figures 7A, 7B, and 7C.

Figure 7A shows that a crack runs across the target plate between the two points of collision. If this crack existed even partially before the second (lower-velocity) collision occurred, the extent of damage caused by this collision at 4.100×10^4^ cm/sec (1,345 ft/sec) may not be representative. In this connection, it should be pointed out that the degree of damage seen in figure 7A was not produced in the collision of hot-pressed alumina target plate No. IV with a mercury drop at 4.276×10^4^ cm/sec (1,403 ft/sec). See figure 6A.

Figures 7B and 7C show that the eye of the collision is again bounded by a circle of short cracks. Less than half of the eye of the collision that occurred at 4.996×10^4^ cm/sec (1,639 ft/sec) can be seen in figure 7C; the remaining part of this area was apparently lost when a chip from the edge of the specimen broke away. Because the eye was broken in half, the crack structure below the surface of the opaque hot-pressed alumina can be observed. Microscopic inspection with the target plate turned at an angle revealed that *the short and more or less parallel cracks that bound the eye run deep into the ceramic*; they may extend through the target plate. *The innermost cracks of the ring may have formed nearly perpendicular to the surface*, *but the outermost cracks formed at an angle estimated roughly to be close to* 45° *to the surface.*

Figure 7A shows that seven radial cracks formed around the eye of the lower-velocity collision; the longer cracks are characterized by dichotomous branching. Comparison of the damage on hot-pressed alumina target plate No. V with that on plate No. IV suggests that the circular cracks that bound the eye of the collision form either with or without the formation of the long radial cracks (see fig. 6). On the other hand, the damage resulting from collision with the drop at 4.100×10^4^ cm/sec (1,345 ft/sec) may be misleading, as was pointed out above. Almost invisible cracks may have been produced in this plate as a result of the edge collision with a mercury drop it had already suffered.

An interesting crack, which appears to reflect the contour of the edge of the target plate, is indicated with an arrow in figure 7A. This crack intersects five of the seven radial cracks that run out from the eye of the lower-velocity collision. The possible origin of this crack is discussed in section 4.2 (e).

## 4. Failure

In order to understand how the observed damage was produced, it is necessary to review the stresses imposed by an impinging liquid drop.

### 4.1. Stresses Produced in Liquid Drop Collisions

When a liquid drop collides with the planar surface of a solid, (a) it exerts a localized pressure and (b) it flows out radially around the central point of impingement.

#### a. Localized Pressure

During the early stages of the collision between a liquid drop and the planar surface of a solid, maximum pressure exists in a ring around the central point of the collision. At the first instant of the collision, when the maximum pressure is higher than at any later time, this ring may be regarded as a point circle. The radius of the ring of maximum pressure increases and the value of the maximum pressure in the ring decreases with time until the radius of the circle of contact between the drop and the solid is about 0.6 of the original radius of the drop. When this stage of the collision is reached, the ring of maximum pressure vanishes.

Savic and Boult [1]^1^ have given a hydrodynamic treatment of the collision of a drop of incompressible liquid with a completely unyielding solid. They found that the pressure is infinite at the first instant of the collision when the ring of maximum pressure may be regarded as a point circle. They state that this infinity is prevented physically by the fact that the high-initial pressure is absorbed in the bulk compressibility of the liquid, giving rise to an emitted compression wave.

According to the treatment of Savic and Boult [1], very high pressure maxima are developed during the early stages of the collision when the pressure maximum exists in a ring around the central point of the collision. Approximate values of the pressure maxima found by them for various values of the radius of the circle of contact between the drop and solid surface are given in table 3. They found that when the radius of the circle of contact is about 0.6 of the original radius of the drop, the ring of maximum pressure vanishes, maximum pressure then exists at the center of the drop, and the pressure profile across the contact area decreases from the center to the periphery of the circle of contact.

In its ability to exert a localized pressure on the surface of a solid with which it collides, an impinging drop acts like a spherical indenter. The stresses that are introduced into an elastic plate by a spherical indenter were studied with the use of small foam rubber pads and spherical indenters of glass and steel. The kinds of stresses that act and the directions in which they act were deduced from the behavior of blemishes (holes and scratches) in the rubber as observed under low-power magnification.

**Figure 8:**
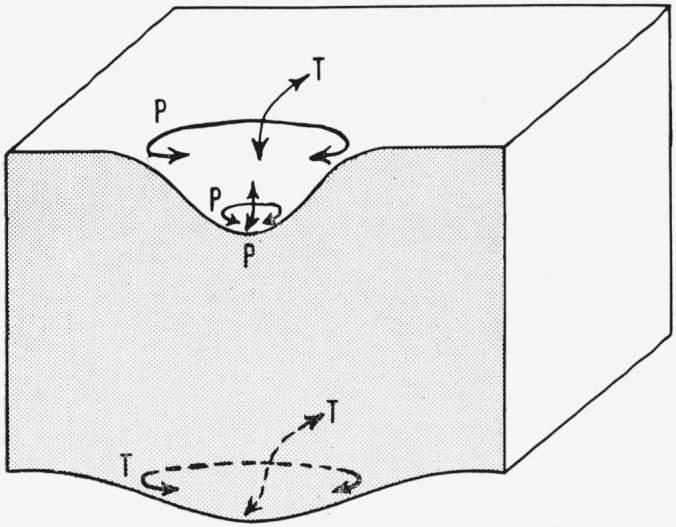
Principal stresses (P, pressure; T, tension) at the concavity and convexity produced by a spherical indenter pressing against an elastic solid plate.

The principal stresses at the periphery of the area compressed by an indenter are tensile and compressive (see fig. 8). This was shown by pressing a steel sphere into a rubber pad. Near the center of the compressed area below an indenter the principal stresses are all compressive (see fig. 8). This was shown by use of a spherical glass indenter flattened on one side; the behavior of the rubber under the indenter as it was pressed into the pad was observed through the flat area on the sphere. The principal stresses at the convexity on the reverse side of a plate into which an indenter is pressed are all tensile (see fig. 8). This was shown by pressing a rubber pad over a steel sphere.

Although an impinging liquid drop acts like a spherical indenter or like an impinging solid sphere in exerting a localized pressure, the pressure it exerts is never as great as that exerted by an impinging solid sphere for any given impingement velocity. This is because part of the collision energy of an impinging drop is transformed into radial flow of the liquid of which it is composed. An impinging solid sphere can inflict damage only by exerting a localized pressure; an impinging liquid drop can inflict damage both by the localized pressure it exerts and by its radial flow.

#### b. Radial Flow

The impact pressure produced by the collision of a liquid drop with the planar surface of a solid drives the liquid that is close to the solid surface radially outward around a central stagnation point. The flow velocity can become very high. In the case of a waterdrop colliding with a glass plate at free-fall velocity, it has been found to approach ten times the value of the impingement velocity for short times after the collision incident [2].

The radially flowing liquid of an impinging drop exerts a shear stress on the surface of the solid over which it is running. The shear stress *τ* between layers of liquid in laminar flow is given by *µ(∂v/∂z*) where *µ* is the viscosity of the liquid, *v* is the velocity at which the liquid is moving, and *z* is the direction through the thickness of the liquid sheet. The layer of liquid molecules in direct contact with the surface of the solid has zero velocity, but the velocity gradient is not zero and the shear stress is applied to the solid.

If the radial flow of an impinging liquid drop runs over a surface protrusion, it exerts forces against the protrusion that tend to separate the protrusion from the underlying layers of material. Pressure exerted against the protrusion by the flowing liquid tends to move the protrusion along the planar surface of the solid and results in a shear stress at the base of the protrusion. The pressure exerted by the liquid also results in a turning moment that tends to bend the protrusion over. If the forces exerted by the rapid flow of liquid are large enough, failure may occur. The protrusion may be bent over, it may be broken off, or part of the solid material below the surface may be torn out with it.

### 4.2 Correlation of Observed Failures With Stresses

#### a. Undamaged Center

The damage marks consisted of an undamaged center surrounded by a polygon of cracks on sapphire and by a circle of cracks on hot-pressed alumina. The undamaged center is the part of the damage mark that was under high pressure during the collision. If a cavity formed during the collision, the undamaged center was the highly compressed material at the bottom of the cavity where the principal stresses are all compressive (see fig. 8). Brittle materials, such as glass, fail in tension [3], and it can be surmised that white sapphire and hot-pressed alumina will fail in tension. Because no tensile stresses form in the compressed solid material near the center of the collision, failure would not be expected here.

#### b. Polygonal or Circular Cracks

The polygonal and circular cracks formed in the region of tensile stress around the central compressed area (see fig. 8). It seems reasonable to suppose that these cracks are the result of a tensile failure.

In the case of a hemispherical distribution of pressure on the surface of a semi-infinite medium, the maximum tensile stress is radial, exists at the periphery of the compressed area, and is given by [3],

$$\sigma_{r}=\left(1-2v\right)/3q_{0}$$

where *v* is Poisson’s ratio and *q*_0_ is the maximum pressure at the center of the compressed area. The type of pressure distribution for which this equation applies exists under an impinging liquid drop only after the circular pressure peaks that develop during the earliest stages of the collision have vanished. This occurs when the radius of the circle of contact is about 0.6 of the original radius of the drop (see sec. 4.1). The maximum pressure at this time according to the hydrodynamic treatment of Savic and Boult [1] for a drop of incompressible liquid impinging against the planar surface of an unyielding solid, is 9.3×10^9^ d/cm^2^ (135,000 psi) for a mercury drop colliding at 3.581×10^4^ cm/sec (1,175 ft/sec). The damage mark made on a white sapphire target plate on collision with a 0.2-cm mercury drop at this velocity is shown in figure 2B. Using this value for the maximum pressure, *g*_0_, and 0.26 for an average value^2^ of Poisson’s ratio, *v*, a value of 1.5×10^9^ d/cm^2^ (22,000 psi) is obtained for the radial tensile stress, *σ_r_.* This is only about half of the tensile strength of white sapphire. See modulus of rupture in table 1. From figure 2B it can be seen that the radius of the area enclosed by the outermost polygonal cracks is about 0.05 cm or half the original radius of the 0.2-cm-diam mercury drop, whereas the calculated value of *σ_r_* is for a radius of about 0.06 cm.

Furthermore, polygonal cracks have formed at distances from the central point of the collision that, range from about 0.025 to about 0.05 cm. From the treatment of Savic and Boult [1], the maximum pressure would be increased by a factor of 2 for the case that the radius of the circle of contact is 0.03 cm, 0.3 of the radius of the original drop, and would be increased by very close to a factor of 3 for the case that the radius of the circle of contact is 0.02 cm, 0.2 of the radius of the original drop. See table 3. Hence it can be surmised that the radial tensile stresses are from 2 to 3 times higher than 1.5×10^9^ d/cm^2^ (22,000 psi) at the distances from the central point of the collision where polygonal cracks are observed to have formed, and are in excess of the tensile strength of white sapphire. However, because the equation given for the radial tensile stress, *σ_r_*, is based on a different pressure distribution from that which exists in a liquid drop when the radius of the circle of contact is less than about 0.6 of the original radius of the drop, this equation cannot validly be used to compute them.

The difference in the type of cracks (polygonal or circular) appears to result from the degree of anisotropy of the two ceramics. The single-crystal white sapphire is characterized by cleavage planes on which failure occurs first whereas polycrystalline hot-pressed alumina is isotropic.

#### c. Breaking Out of Material

White sapphire is eroded away along the polygonal cracks that formed in it. This damage appears to be the result of the radial flow of the mercury drops that impinged. If the broken edges of the cracks were raised above the planar surface of the target plate by plastic flow, they may have provided protrusions against which the radially flowing mercury exerted pressure. The pressure would result in a shear stress below the protrusion and a turning moment exerted against it, which may have caused the loss of material.

The reasoning just given is supported by the observation that the loss of sapphire material occurred on the side of the crack away from the central point of the collision. If the raised broken edge of the crack provided a protrusion against which the radial flow of the mercury of the drop could exert a pressure, this is the side of the crack against which the pressure would be exerted.

There are two other observations that support the reasoning given. One of these is that the outermost polygonal cracks are more eroded than the innermost. This may be explained in terms of the magnitude of the radial flow velocity at different distances from the stagnation point (zero flow velocity) at the center of the collision. On the other hand, there may have been an increase in the magnitude of the radial tensile stress with increase in distance from the center of the collision and the outermost cracks may, therefore, have developed higher raised edges due to plastic flow. The other observation is that the erosion damage increased in severity as the impingement velocity was increased. This could be in agreement with the fact that the velocity of the radial flow is increased as the impingement velocity is increased. However, the magnitude of the radial tensile stresses introduced is also increased as the impingement velocity is increased and this could explain the observation in terms of higher raised edges along the cracks.

#### d. Radial Cracks

Radial cracks formed at the highest impingement velocities at which mercury drops struck the target plates of both white sapphire and hot-pressed alumina. They ran out from the center of the collision and extended completely across and through the target plates.

These cracks are probably also the result of a tensile failure. Tensile stresses perpendicular to the radial principal planes of stress are required to account for them. The stresses perpendicular to the radial principal planes of stress around the periphery of the area of the target plate compressed by the colliding drop are compressive (see sec. 4.1 and fig. 8).

It is possible that these cracks may originate on the reverse face of the target plate if the compressional wave initiated by the collision reflects appreciably as a tension wave.^3^ The principal stresses that exist on the reverse face of the plate if this reflection occurs are all tensile stresses (see fig. 8). Hence radial cracks could form on the reverse face of the plate as a result of a tensile failure. It is possible that the radial cracks originate on the reverse face of the target plate and progress through it to the collision surface.

#### e. Curved Cracks

Cracks that reflect the curvature of the bounding edge of the target plates formed in both white sapphire and hot-pressed alumina. A wave of compression is initiated in the target plate as a result of the collision. When this wave reaches the curved edges of the target plate, it reflects both as a tension wave and as a transverse wave. It seems very likely that the curved cracks formed when the level of stress in the reflected tension wave exceeded the tensile strength of the ceramic material.

From figures 5A and 7A it can be seen that the distance from the edge of the hot-pressed alumina target plate to the curved crack is about the same as the distance from the edge of the white sapphire target plate to the curved crack that formed furthest from the edge of it. On the basis of the observation that no second crack formed in hot-pressed alumina, and of the information that the tensile strengths of the ceramics and the sizes and velocities of the impinging drops were closely similar, it can be deduced that the crack furthest from the edge of the white sapphire target plate formed first. This deduction is in agreement with the observation that the curvature of the two cracks is in the same direction. If the crack nearest the edge of the sapphire target plate had formed first, the compressional wave initiated by the collision would have begun to reflect from the curved surface of this crack, and it would be expected that, if the level of tensile stress in the tension wave reflected from this crack again exceeded the tensile strength of the ceramic, the second crack to form would curve in the opposite direction.

The curved edges of the target plates focus the reflected tension waves in the same way that a curved mirror focuses reflected light waves. This is in agreement with the observation that the curved cracks are isolated; they do not extend from edge to edge of the target plate. The curved cracks formed in white sapphire are below the surface. This may be ascribed to the effect of the bevelled edge of the target plate on the reflected wave. In hot-pressed alumina, however, the curved crack extends to the surface.

If the curved crack furthest from the edge of the white sapphire target plate formed first, as seems to be the case from the evidence at hand, the mechanism by means of which the crack nearest the edge of the target plate then formed needs to be explained.

At the time that the curved crack furthest from the edge of the target plate forms, a tension wave superposed on a pressure wave is trapped between this curved crack and the curved edge of the target plate. The curvature of both bounding surfaces between which the trapped wave must travel is such as to focus the wave further. This is in agreement with the observation that the crack that eventually forms closest to the edge of the target plate is shorter than the crack that appeared first and that formed furthest from the edge of the target plate. If the boundaries between which the trapped wave must reflect were straight, the level of tensile stress in the tension wave would never again exceed the tensile strength of the ceramic. In the case of reflection between curved boundaries, the focusing effect of the boundaries may make the formation of the second fracture possible.

## 5. Resistance to Waterdrop Impingement

The information of greatest interest from a practical standpoint is the resistance of white sapphire and of hot-pressed alumina to waterdrop impingement. This information was not obtained in the tests because impingement velocities high enough to fracture these ceramics on colliding with a waterdrop were not reached. In this section an equation is developed from which an estimate of the resistance of these ceramics to waterdrop impingement can be obtained by extrapolation of their resistance to mercury drop impingement.

To develop this equation the following assumptions are made: (1) A concavity (no matter how small) forms in a thin ceramic plate, whose reverse face is a free surface, as a result of high-speed liquid-drop impingement; (2) elastic recovery without fracture occurs when the impingement velocities are low and the depth of the concavity is small; and (3) corresponding velocities to produce equal depth of concavity in a given edge-supported ceramic plate by impingement with drops of a liquid A and drops of a liquid B are corresponding velocities for fracture when the concavity reaches the depth at which the tensile stresses that develop around it (either by themselves or when they are reinforced by the tension wave reflected from the reverse face of the plate) are sufficient to produce fracture.

Assumptions (1) and (2) are reasonable because, although microscopic plastic deformation at stress concentrations has been found to take place in white sapphire and ruby [4], in macroscopic terms these ceramics may be said to yield elastically until fracture occurs. The validity of assumption (3) is open to question. It depends on similarity considerations and, therefore, on the implication that concavities of equal depth produced by impingement of drops of different liquids are of similar shape. However, it may be sufficiently reliable for a rough estimate if the diameters of the drops and the viscosity and surface tension of the liquids used are not too widely different. Within the validity of the assumptions made, an equation from which extrapolated corresponding velocities for equal fracture of thin edge-supported ceramic plates by liquid-drop impingement can be calculated, has been developed as follows.

When a fast-moving target plate collides with a stationary liquid drop, a core of material through the target plate under the collision area is slowed down with respect to the remainder of the target plate as a result of the collision. As in a previous treatment [5], the situation is idealized in two ways. First, the core is regarded as a true cylinder that is free to move in the direction of the collision blow but is restrained laterally. A similar cylinder exists in the liquid of which the drop is composed. Secondly, the compressional waves that move through the cylinder in the target plate and through the cylinder in the drop are regarded as plane waves.

The depth, *δ′*, of the concavity that forms elastically in a ceramic target plate as a result of highspeed collision with a liquid drop is proportional both to the particle velocity, *v′*, produced in the core of target material under the contact area as a result of the collision, and to the time, *t*, that this particle velocity exists. Then,

δ′=kv′t (1)

where *k* is a constant. It has been shown [6] that, for the case that the target plate collides with a liquid drop rather than with a solid sphere,

$$v\prime =\frac{\alpha zV}{z\prime +\alpha z}$$ (2)

where *α* is a coefficient having a value less than one, *V* is the impingement velocity, and *z=c_ρ_* where *ρ* is the density and *c* is the speed of irrotational waves in infinite medium. Primed quantities refer to the material of the solid target plate; unprimed quantities refer to the liquid of which the drop is composed. All quantities are in cgs units. It has also been shown [6] that

α=0.41/[1+(0.59z/z′)]. (3)

As before [5], the time *t* is the time required for the compressional wave initiated in the liquid of the drop to move through the drop, reflect as a tension wave from the free liquid-to-air interface, and return to the contact area between the drop and the target plate. Then,

t=2d/c (4)

where *d* is the diameter of the drop.

By substituting eq (2) and (4) into (1), the depth of concavity produced by collision of the target plate with a drop of liquid *A* is

$$\delta_{A}^{\prime }=k\left(\frac{\alpha_{A}z_{A}V_{A}}{z\prime +\alpha_{A}z_{A}}\right)\left(\frac{2d_{A}}{c_{A}}\right).$$ (5)

Similarly, for a drop of a liquid *B*,

$$\delta_{B}^{\prime }=k\left(\frac{\alpha_{B}z_{B}V_{B}}{z\prime +\alpha_{B}z_{B}}\right)\left(\frac{2d_{B}}{c_{B}}\right).$$ (6)

By equating eq (5) and (6), it is found that

$$V_{A}=\left(\frac{\alpha_{B}z_{B}}{\alpha_{A}z_{A}}\right)\left(\frac{z\prime +\alpha_{A}z_{A}}{z\prime +\alpha_{B}z_{B}}\right)\left(\frac{c_{A}}{c_{B}}\right)\left(\frac{d_{B}}{d_{A}}\right)V_{B}.$$ (7)

If fracture of the ceramic occurs when the concavity that forms reaches a certain depth, then, by substituting into eq (7) the velocity known to produce fracture on a brittle elastic solid by impingement of drops of a liquid *B*, the velocity required to produce fracture of the same solid by impingement of drops of a liquid *A* can be calculated.

Let liquid *A* be water, liquid *B* be mercury, and the target plate be of white sapphire. From eq (3), and the data of table 4, the coefficient *α* for waterdrops impinging against white sapphire is 0.40, and the coefficient *α* for mercury drops impinging against white sapphire is 0.32. Then, from eq (7) and the data of table 4,

$$V_{W}=9.6\left(\frac{d_{M}}{d_{W}}\right)V_{M},$$ (8)

where sub-*W* indicates water and sub-*M* indicates mercury. For the condition that the waterdrop and mercury drop have the same diameter, *V_W_*=9.6 *V_M_.* It required an impingement velocity of 3.514×10^4^ cm/sec (1,153 ft/sec) to produce fracture of a white sapphire target plate 0.318 cm (0.125 in.) thick in collision with a 0.2-cm mercury drop (see fig. 1). From eq (8), a 0.2-cm waterdrop would have to collide with the same target plate at a velocity of 33.7×10^4^ cm/sec (11,100 ft/sec) in order to produce fracture. Taking the speed of sound in air at 0° C to be 3.317×10^4^ cm/sec (1,088 ft/sec), the Mach Number equivalent is 10.

*Experimental verification of eq* (7) *is needed before corresponding velocities calculated by use of it can be relied upon.* The only experimental data available to check eq (7) are those being obtained in a study currently in progress on the fracture of an unidentified polycrystalline alumina by impingement of polyethylene pellets [7]. In this study, 12.7-cm (5-in.) square plates of 99+ percent pure polycrystalline alumina 0.635 cm (0.25 in.) thick are being used as targets for impingement of 0.5-cm polyethylene pellets fired at a constant velocity of 23.5×10^4^ cm/sec (7,700 ft/sec). The ceramic plates are maintained at a temperature of 1,371 °C (2,500 °F) during the firings. The angle at which the polyethylene pellets strike the target plates is increased until erosion, and finally breakage failure, occurs. The data available at present are for two plates tested to complete failure. Significant erosion was not noted until the impact angle was in each case 40°. One of the plates failed to the point of breakage at an impact angle of 40° and the other plate failed to the same extent at an impact angle of 60°. These impact angles were reported to correspond to normal velocities of 14.9×10^4^ cm/sec (4,900 ft/sec) and 20.4×10^4^ cm/sec (6,700 ft/sec), respectively [7].

Because the resistance of white sapphire and of hot-pressed alumina to mercury-drop impingement was found to be nearly the same, and because the more common, cream-white polycrystalline alumina is a ceramic of comparable strength properties, the reliability of eq (7) can be tested roughly by calculating the velocity required to damage white sapphire to the point of radial crack formation by impingement of a 0.5-cm polyethylene pellet.

By use of eq (3) and the data 4 table 4, it is found that the coefficient *α* for collision of a polyethylene pellet with a white sapphire plate is 0.40. By use of eq (7) and the data of table 4, it is found that

$$V_{P}=10.5\left(\frac{d_{M}}{d_{P}}\right)V_{M}$$ (9)

where the sub-*P* notation indicates polyethylene. From figure 4 it can be seen that radial fracture of a white sapphire target plate occurred as a result of impingement of a 0.2-cm mercury drop at a velocity of 4.420×10^4^ cm/sec (1,450 ft/sec). Using this velocity for *V_M_*, 9.2 cm for *d_M_*, and 0.5 cm for *d_P_*, eq (9) predicts that an impingement velocity of 18.6×10^4^ cm/sec (6,090 ft/sec) would be required to produce the extent of fracture seen in figure 4 with use of a 0.5-cm polyethylene pellet.

The velocity calculated by use of eq (9) is between the two values that have been found experimentally. However, the fact that the ceramic target plates were held at a temperature of 1,371 °C (2,500 °F) during the test firings, which may have affected their resistance, the fact that the polyethylene pellets impinged at an angle, which should result in an unsymmetrical distribution of stress, and the fact that the drop size was considerably different raise questions that reduce the significance of this good agreement.

Jackman and Roberts [8] have found that the strength of poly crystalline alumina is not changed by an increase in temperature until the temperature reaches 700 °C (1,292 °F). If the temperature is increased further, there is a gradual decrease of strength with increase of temperature [8]. At 1,300 °C (2,372 °F) the strength is roughly one-third of its room-temperature value [8]. The strength of white sapphire has a very different temperature dependence [5, 8]. It decreases with increase of temperature to a minimum in the range of 300 to 600 °C (572 to 1,112 °F) and then increases with further increase in temperature until, at about 1,000 to 1,200 °C (1,832 to 2,192 °F), it is very close to its room-temperature value.

It is possible that, at the high rates of loading involved in the impingement tests, the temperature of the ceramic plates may not have an important effect on the velocity that an impinging pellet must have to break them.^4^ But this effect, the effect of drop size, and the effect of the angle at which the collision occurs need to be determined.

Until eq (7) is verified more conclusively with experimental evidence, *corresponding velocities calculated by use of it should not be regarded as more than estimates*.

## 6. Ultimate Failure

In order to extrapolate from the observed liquid-drop-impingement failure of white sapphire and hot-pressed alumina to what may be expected at higher collision velocities, it is useful to consider the failure of low-strength brittle solids, such as poly(methyl methacrylate) and polystyrene, under imposed stresses of the same kind.

Polygonal cracks have been observed in poly (methyl methacrylate) as a result of waterdrop impingement at a velocity of 2.234×10^4^ cm/sec (733 ft/sec) [9]. The cracks were widened by breaking out of the surface material along them in a direction away from the central point of impact as a result of the radial flow of the water. With respect to these features, the damage marks produced on low-strength poly (methyl methacrylate) by waterdrops impinging at a velocity of 2.234×10^4^ cm/sec (733 ft/sec) are similar to the damage marks produced on high-strength white sapphire by mercury drops impinging at a velocity of 3.514×10^4^ cm/sec (1,153 ft/sec).

**Figure 9:**
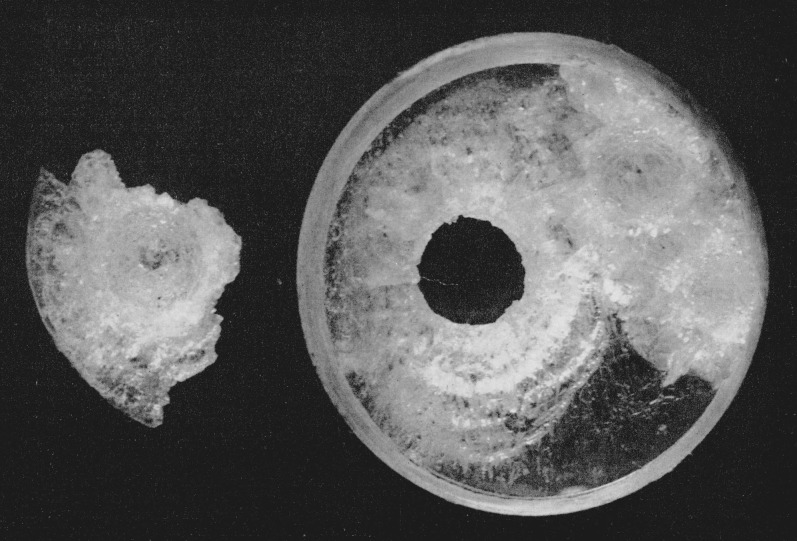
Polystyrene target plate that collided with waterdrops at a velocity of 7.666×10^4^ cm/sec (2,515 ft/sec). The plug of polystyrene cut out of the target plate is also shown. (Magnification × 6.1)

The ultimate kind of failure that white sapphire will undergo may be predicted from the observed damage done to a plate of polystyrene 0.318-cm (0.125-in.) thick that collided with a waterdrop at a velocity of 7.666×10^4^ cm/sec (2,515 ft/sec). A circular plug of polystyrene was cut out of the plate as a result of the collision (see fig. 9). It can be expected that similar damage will be done to white sapphire and to hot-pressed alumina as a result of collision with a waterdrop. The only difference will be that the collision velocity required to produce this degree of damage will be much higher for the high-strength ceramics than for the relatively weak polystyrene. Indeed, it has already been reported informally [7] that a plug has been cut out of a plate of polycrystalline alumina as a result of collision with a polyethylene pellet.

## Figures and Tables

**Table 1:** Physical properties of white sapphire and hot-pressed alumina

Material	Hot-pressed alumina^a^	White sapphire^b^
		
Density g/cm^3^	3.95	$\begin{array}{l} \;3.98\\ \left(3.4\;\text{to}\;3.8\right)\times 10^{12}\\ \left(50\;\text{to}\;55\right)\times 10^{6} \end{array}\}\text{depending on position of crystal C-axis}$.
Modulus of elasticity		
d/cm^2^	3.4×10^12^	
psi	50×10^6^	
Modulus of rupture c		
d/cm^2^	2.4×10^9^	$\begin{array}{l} \left(2.8\;\text{to}\;9.0\right)\times 10^{9}\\ \left(40\;\text{to}\;130\right)\times 10^{3} \end{array}\}\text{at}\;30{}^{\circ}C.$
psi	35×10^3^	
Compressive strength		
d/cm^2^	2.8×10^10^	$\begin{array}{l} 2.1\times 10^{10}\\ 300\times 10^{3} \end{array}\}\text{at}\;25\;{}^{\circ}C.$
psi	400×10^3^	
Knoop hardness (K 100)	2,000	1,525 to 2,000
Mohs hardness	9 to 9.5	9
Melting point °C	2,000	2,040

a Tabulated data supplied by Norton Co. for H. P. Alundum. H. P. Alundum is hot-pressed alumina of uniform density and hardness manufactured by sintering the pure aluminum oxide crystals; no bonding matrix is used.

b Data supplied by Linde Air Products Co.

c The modulus of rupture (flexural strength) is probably 20- to 30-percent higher than the tensile strength of these ceramic materials; measurement of their true tensile strength is complicated by factors related to their brittleness and the difficulty in alining the specimens.

**Table 2:** Data for collisions of target plates of white sapphire and hot-pressed alumina with 0.2-cm-diam drops

Specimen	Firing	Drop liquid	Collision velocity^a^		Effect on target plate

Target plates of white sapphire					

			*10*^4^ *cm/sec*	*ft/sec*	
I	$\{\begin{array}{c} a\\ b\\ c \end{array}$	water	4.779	1, 568	No damage.
		water	6.620	2, 172	No damage.
		water	7.529	2, 470	Missed waterdrop; target plate cracked due to firing shock.
II	$\{\begin{array}{c} a\\ b\\ \begin{array}{l} c\\ d \end{array} \end{array}$	mercury	2.890	948	No damage.
		mercury	3.197	1,049	No damage.
		mercury	3.216	1,055	No damage.
		mercury	3.514	1,153	Damaged; see fig. 1.
III	……	mercury	3.581	1,175	Damaged; see figs 2 and 3.
IV	……	mercury	4.420	1,450	Damaged; see fig. 4.
V	……	mercury	4.584	1,504	Damaged; see fig. 5.

Target plates of hot-pressed alumina					

I	……	water	7.346	2,410	No conclusive evidence of damage.
II	……	water	9.144	3,000	Target plate shattered due to to firing shock.
III	……	water	11.88	3,898	No conclusive evidence of damage.
IV	……	mercury	4.276	1,403	Damaged; see fig. 6.
V	$\left\{ \begin{array}{l} a\\ b \end{array}\right.$	mercury	4.996	1,639	Damaged by edge collision; see figs. 7A and 7C.
		mercury	4.100	1,345	Damaged; see figs. 7A and 7B.

a The velocity data were supplied by Donald E. Hurd, Convair, Division of General Dynamics Corp., San Diego, Calif.

**Table 3:** Maximum pressure in an impinging liquid drop^a^

Radius of circle of contact	Approximate value of maximum pressure
	
0.1 *R*	3 *_ρ_U*^2^
.2 *R*	32ρU2
.3 *R*	*_ρ_U*^2^
………	………
~0. 6 *R*	½ *_ρ_U*^2^

a *R* is the original radius of the drop, *ρ* is the density of the liquid of which the drop is composed, and *U* is the impingement velocity.

**Table 4:** Acoustic impedance

Material	Density	Sound speed	Acoustic impedance
			
	*g/cm*^3^	*cm/sec*	*g*/(*cm^2^·sec*)
Water	a0.99707	b1.497×10^5^	0.1493×10^6^
Mercury	a13.546	b1.451×10^5^	1.966×10^6^
Polyethylene	a0.92	c1.95×10^5^	0.1794×10^6^
White sapphire	d3.98	f11.00×10^5^	4.378×10^6^
Hot-pressed alumina	e3.95	f11.00×10^5^	4.345×10^6^

a Data from Handbook of Chemistry and Physics.

b Data from L. Bergmann, Der Ultraschall, S. Hirzel, Stuttgart, 1954.

c Data from American Institute of Physics Handbook, 1958.

d Data supplied by Linde Air Products Company.

e Data supplied for H. P. Alundum by Norton Company.

f Approximate average value supplied by John B. Wachtman, Jr., of NBS Engineering Ceramics Section.

## Footnotes

1 Figures in brackets indicate the literature references at the end of this paper.

2 This average value of Poisson’s ratio for white sapphire and polycrystalline alumina was kindly supplied by J. B. Wachtman, Jr., of NBS Engineering Ceramics Section.

3 The extent to which reflection and transmission of the compressional wave may have occurred at the adhesive bond between the ceramic target plate and the aluminum backing plate is not known.

4 I am indebted to J. B. Wachtman, Jr. of NBS Engineering Ceramics Section for this suggestion.
